# Confidential Cooperative Communication with the Trust Degree of Jammer

**DOI:** 10.3390/e21060595

**Published:** 2019-06-15

**Authors:** Mingxiong Zhao, Di Liu, Hui Gao, Wei Feng

**Affiliations:** 1 National Pilot School of Software, Yunnan University, Kunming 650504, China; 2 Key Laboratory in Software Engineering of Yunnan Province, Kunming 650504, China; 3 School of Information and Communication Engineering, Beijing University of Posts and Telecommunications, Beijing 100876, China; 4 School of Communication Engineering, Hangzhou Dianzi University, Hangzhou 310018, China

**Keywords:** trust degree, confidential communication, cooperative jamming, transmit SNR

## Abstract

In this paper, we consider the trust degree of a jammer, defined as the probability that the jammer cooperates to secure the legitimate transmission, and investigate its influence on confidential cooperative communication. According to the trust degree, we derive the closed-form optimal transmit signal-to-noise ratio (SNR) of the confidential message, $\rho_{c}^{⋆}$, to maximize the expected secrecy rate, and further obtain the relationship between $\rho_{c}^{⋆}$ and the trust degree associated with the transmit SNR at the transmit user and channel gains. Simulation results demonstrate that the trust degree has a great effect on the transmit SNR of the confidential message and helps improve the performance of confidential cooperation in terms of the expected secrecy rate.

## 1. Introduction

With the rapid growth of communications between socially-connected users, not only physical parameters, e.g., channel qualities and traffic demands, but also the social relationship among users, such as kinship, friendship, and acquaintance, have been taken into account as key design parameters for efficient cooperative communications [1,2,3,4,5]. As the most fundamental concept, the trust degree of the social relationship is interpreted as the degree that reveals how willingly a node helps other nodes’ communication in cooperative communications [5]. In other words, the node would like to cooperate with others by consuming their own resources if they have a close relationship, i.e., a high trust degree, which can be evaluated and quantified based on the previous direct or indirect information according to observations of behavior [6].

In the literature, the trust degree has triggered plenty of research interest in various fields, including content caching, mobile social communications, and especially cooperative communications [1,2,3,4,5]. More specifically, the optimal social-aware relay selection strategy was proposed in [1], while a partner selection algorithm was designed based on the social-position relationship in [2]. The trust degree-based beamforming and transmission strategies were investigated for one relay in [3] and two relays with different trusts in [4] for a MISO cooperative communication system. Meanwhile, the traffic demand-driven user cooperation strategies were considered for various antenna configurations based on the trust degree in [5].

Due to the broadcasting nature of wireless mediums, physical (PHY)-layer security has also drawn significant attention in communication networks [7,8,9,10,11]. Specifically, the authors in [8] gave a constructive survey on threats and attacks on mobile networks, while a comprehensive survey on cooperative relaying and jamming strategies for physical layer security was presented in [9]. Meanwhile, the performance analysis of mmWave-overlaid microwave cellular networks was given in [10] from the security perspective, and secrecy analysis with passive eavesdroppers by using partial cooperation was investigated in [11]. Furthermore, with the rapid development of social awareness networks, the combination of the trust degree and PHY-layer security with application to cooperative communications has attracted plenty of research [12,13,14,15]. To be specific, the authors in [12] treated potential eavesdroppers as relays to transmit messages and maximize the expected secrecy rate according to their trustworthiness, instead of regarding them as wiretappers all the time. In [13,14], trust degree-based cooperative secure transmission strategies were proposed, and users with sufficiently high trust degrees would cooperate to transmit data or jamming signals. In [15], jamming-aided cooperative cooperation based on trust degree was investigated to maximize the security rate of the data transmission for P2P communications, where the authors applied a brute-force approach to search over all possible power allocations of the confidential message and jamming signal. However, the trust degree in [13,14] was exploited to choose trustworthy users (jammer [13], jammer and relay [14]) and filter out untrustworthy users (dummy nodes), and the explicit relationship between power allocations and trust degree had not been investigated and given in [15]. Therefore, the characteristics of the jammer’ trust degree were not fully utilized to effectively design cooperative secure transmission strategies, and the insight into how the trust degree affects the confidential cooperation was not explicitly investigated and well presented.

Motivated by the aforementioned research and problems, we have observed that the trust degree plays a significant role in cooperative secure communications. Hence, in this paper, we consider the probability that the jammer cooperates in secure legitimate transmission as its trust degree and investigate its influence on the performance of confidential cooperative communications. Different from [15], we not only consider the transmit signal-to-noise ratios (SNRs) of the confidential message and jamming signal, but also investigate the influence of artificial interference at the transmit user (Tu) in the case that the jamming signal from the jammer is not sufficient when the channel gain between the jammer and eavesdropper is weak and obtain their optimal transmit SNRs in closed-form to maximize the expected secrecy rate according to the trust degree. Meanwhile, we further achieve some meaningful results based on the relationship among the trust degree, channel gains, and the transmit SNR at Tu.

Notation: For a complex scalar x, its complex conjugate is denoted by $\bar{x}$. E[·] and CN(·) denote the statistical expectation and complex Gaussian distributions, respectively.

## 2. Trust Degree and System Model

### 2.1. Trust Degree

With the rapid growth of online social networks, more and more people are getting involved in online social interactions. Therefore, the social relationship has emerged as an important issue to investigate how the degree of closeness of the social relationship between users affects their communication strategies [5,16,17,18]. In the cooperative communication systems, the trust degree can be interpreted as the degree that reveals how much a node is willing to help the communication of the other node [1,3,5]. Similarly, in our system model, the trust degree between T and J, α, is defined by the probability that J helps to secure the transmission between T and R, and thus, α is a value in the range of 0≤α≤1.

In the literature, the trust degree has been evaluated and quantified in various ways [6,19,20,21,22,23]. In emergency networks such as disaster relief and public safety networks, the most trustworthy nodes would be those in the immediate region, so the trust degree can be measured based on the proximity between nodes, e.g., physical distance [19]. In general mobile networks, the trust degree can be evaluated by the observations of the previous behaviors of the node [6,20,21,22,23]. In [22,23], the trust degree was determined using the Bayesian framework. In the Bayesian framework, the trust degree is given by the ratio of the observations of the positive behavior among total observations, where the positive behavior is that the node behaves in the predefined way of the network. Similar to [23], in this paper, the positive behavior is defined by jammer, which helps to secure the transmission of Tu, and hence, Tu can estimate the trust degree based on the historical observations of the positive behavior of jammer. The trust degree can also be updated according to new observations. However, when the number of observations is sufficiently large, the trust degree will have ignorable change according to new observation, and it will be more like a constant. Therefore, in our system model, we assume that the trust degree remains unchanged during the transmission [5].

### 2.2. System Model

**Figure 1:**
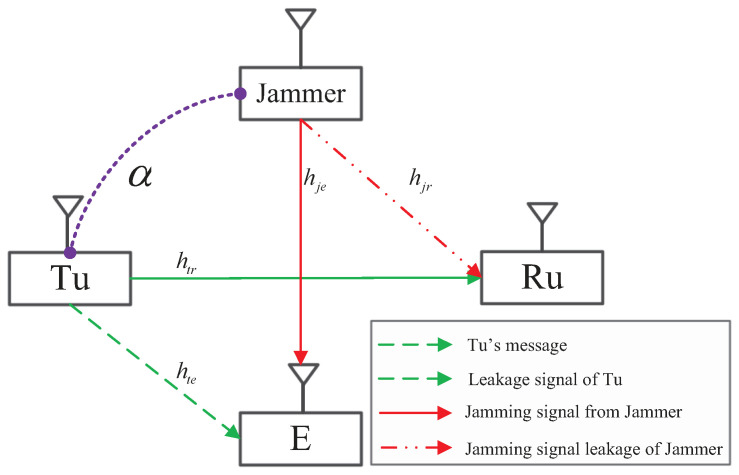
System model. Tu, transmit user; Ru, receive user; E, Eavesdropper.

Consider a user cooperation network as shown in Figure 1, where there are four single-antenna nodes, including a Tu, a receive user (Ru), a jammer, and an eavesdropper, and we denote them by T, R, J, and E, respectively. The channels from T to R and E, and from J to E and R are denoted by $h_{tr}$, $h_{te}$, $h_{je}$, and $h_{jr}$, where all of them follow a complex Gaussian distribution with zero mean and different covariances, $\sigma_{h_{tr}}^{2}$, $\sigma_{h_{te}}^{2}$, $\sigma_{h_{je}}^{2}$ m and $\sigma_{h_{jr}}^{2}$, respectively.

In this paper, we consider that J will transmit the jamming signal to confound E in order to help the legitimate transmission between T and R based on trust degree α, which characterize the willingness that J cooperates with T for secure communications, i.e., J helps with high probability when the trust degree is high. In this system, we consider the following two transmission strategies for confidential communications:

(a) *Direct transmission*: J does not help to confound E with jamming signal $x_{j}$, and thus, T transmits artificial interference, $x_{i}$, together with a confidential message, $x_{c}$, to keep its transmission secure, where $x_{i}$ and $x_{c}$ are independent. In this strategy, the transmit data at T and J are written as $\left\{x_{t}=\sqrt{P_{c}}x_{c}+\sqrt{P_{i}}x_{i},x_{j}^{d}=0\right\}$, while $E[x_{i}{\bar{x}}_{i}]=1$ and $E[x_{c}{\bar{x}}_{c}]=1$, where $P_{c}+P_{i}\leq P_{T}$ and $P_{T}$ is the maximum transmit budget at T.

(b) *Cooperative transmission*: J transmits jamming signal $x_{j}$ to guarantee additionally the secure transmission between T and R and helps to reserve more power for the confidential message at T. However, T also needs to generate artificial interference for this strategy in case the jamming signal from the jammer is not sufficient when channel gain $|h_{je}{|}^{2}$ is small. Thus, the transmit data at T and J are given by $\left\{x_{t}=\sqrt{P_{c}}x_{c}+\sqrt{P_{i}}x_{i},\;x_{j}^{c}=\sqrt{P_{j}}x_{j}\right\}$, while $E[x_{j}^{c}{\bar{x}}_{j}^{c}]=1$, where $P_{j}\leq P_{J}$ and $P_{J}$ is the maximum transmit budget at J.

According to the above strategies, the expected secrecy rate with respect to (w.r.t.) trust degree α, the similar structure of which was given in [15], is obtained by:

(1) $$\begin{array}{c} \bar{R}{}_{se}\left(\rho_{c},\rho_{i},\rho_{j}\right)=\bar{\alpha }{\left[\log_{2}\left(1+\rho_{c}g_{tr}\right)-\log_{2}\left(1+\frac{\rho_{c}g_{te}}{\rho_{i}g_{te}+1}\right)\right]}^{+}\\ +\alpha {\left[\log_{2}\left(1+\rho_{c}g_{tr}\right)-\log_{2}\left(1+\frac{\rho_{c}g_{te}}{\rho_{i}g_{te}+\rho_{j}g_{je}+1}\right)\right]}^{+}, \end{array}$$

in which we assume that $x_{i}$ and $x_{j}$ are prior-known at R, and R can completely cancel $x_{i}$ and $x_{j}$ from its received signal, which is a common assumption used in [12,24]. Notice that the information of jamming and artificial interference can be shared through an alternative wired connection between the transmitter or jammer and the receiver. In (1), ${\left[x\right]}^{+}=\max\left\{0,x\right\}$ and $\bar{\alpha }≜1-\alpha$. The channel gains are defined as $g_{tr}=|h_{tr}{|}^{2},g_{te}={\left|h_{te}\right|}^{2}$ and $g_{je}={\left|h_{je}\right|}^{2}$. The transmit SNRs are given as $\rho_{c}=\frac{P_{c}}{\sigma^{2}},\rho_{i}=\frac{P_{i}}{\sigma^{2}}$ and $\rho_{j}=\frac{P_{j}}{\sigma^{2}}$, where $\sigma^{2}$ is the variance of the complex Gaussian distribution, $\mathrm{CN}(0,\sigma^{2})$, associated with the additive white Gaussian noise (AWGN) at R and E. The first term of (1) denotes the secrecy rate at R obtained by the direct transmission strategy when J does not help with the possibility $\bar{\alpha }$, while the second term represents the secrecy rate achieved by the cooperative transmission when J helps to jam with the possibility α.

## 3. Problem Formulation and Solution

From (1), it is noted that for given $\left\{\rho_{c},\rho_{j}\right\}$, (1) is a monotonic increasing function w.r.t. $\rho_{i}$, and thus, the optimal transmit SNR of artificial interference that maximizes (1) is achieved when $\rho_{i}=\rho_{T}-\rho_{c}$ i.e., $P_{i}=P_{T}-P_{c}$, where $\rho_{T}=\frac{P_{T}}{\sigma^{2}}$. Meanwhile, it also applies to the case that for given $\left\{\rho_{c},\rho_{i}\right\}$, the optimal transmit SNR of the jamming signal is $\rho_{j}^{⋆}=\rho_{J}≜\frac{P_{J}}{\sigma^{2}}$, which maximizes (1). Therefore, the problem that maximizes (1) can be formulated as:

(2a) $$\begin{array}{cc} P:\;\max_{\rho_{c}}\; & {\bar{R}}_{se}\left(\rho_{c}\right) \end{array}$$

(2b) $$\begin{array}{cc} s.t.\; & 0\leq \rho_{c}\leq \rho_{T}, \end{array}$$

where:

(3) $$\begin{array}{c} {\bar{R}}_{se}\left(\rho_{c}\right)=\bar{\alpha }{\left[\log_{2}\left(1+\rho_{c}g_{tr}\right)-\log_{2}\left(1+\frac{\rho_{c}g_{te}}{\left(\rho_{T}-\rho_{c}\right)g_{te}+1}\right)\right]}^{+}\\ +\alpha {\left[\log_{2}\left(1+\rho_{c}g_{tr}\right)-\log_{2}\left(1+\frac{\rho_{c}g_{te}}{\left(\rho_{T}-\rho_{c}\right)g_{te}+\rho_{J}g_{je}+1}\right)\right]}^{+}. \end{array}$$

To maximize the expected secrecy rate in P, the optimal transmit SNR of confidential message, $\rho_{c}^{⋆}$, at T can be achieved resorting to the following theorem.

**Theorem** **1.** *For given channel conditions and transmit SNRs, $\rho_{T}$ and $\rho_{J}$, the optimal transmit SNR of confidential message, $\rho_{c}^{⋆}$, that maximizes the expected secrecy rate at R is obtained as:*

(4) $$\begin{array}{c} \rho_{c}^{⋆}=\left\{\begin{array}{cc} \rho_{1}, & \mathrm{if}\;g_{te}\geq g_{tr},\;\left\{\begin{array}{c} {\left[\frac{\rho_{T}g_{tr}g_{te}+g_{tr}-g_{te}}{g_{tr}g_{je}}\right]}^{+}<\rho_{J}\leq \frac{g_{te}-g_{tr}}{g_{tr}g_{je}}\\ \max\left\{\frac{g_{te}-g_{tr}}{g_{tr}g_{je}},\frac{\rho_{T}g_{tr}g_{te}+g_{tr}-g_{te}}{g_{tr}g_{je}}\right\}<\rho_{J}\leq \frac{\rho_{T}g_{tr}g_{te}+g_{te}-g_{tr}}{g_{tr}g_{je}} \end{array}\right.\\ \rho_{c}^{l}, & \mathrm{if}\;g_{te}\geq g_{tr},\;\frac{g_{te}-g_{tr}}{g_{tr}g_{je}}<\rho_{J}\leq \frac{\rho_{T}g_{tr}g_{te}+g_{tr}-g_{te}}{g_{tr}g_{je}}\\ \rho_{T}, & \mathrm{if}\;g_{te}\geq g_{tr},\;\frac{\rho_{T}g_{tr}g_{te}+g_{te}-g_{tr}}{g_{tr}g_{je}}<\rho_{J}\\ \rho_{2}\;\mathrm{or}\;\rho_{3}, & \mathrm{otherwise} \end{array}\right., \end{array}$$

*where $\rho_{c}^{l}=\frac{\rho_{T}g_{tr}g_{te}+g_{tr}-g_{te}}{g_{tr}g_{te}}$, $\rho_{1}=\frac{\rho_{T}g_{tr}g_{te}+\rho_{J}g_{tr}g_{je}+g_{tr}-g_{te}}{2g_{tr}g_{te}}$,*

$$\begin{array}{c} \rho_{2}=\left\{\begin{array}{c} \min\left\{{\left[\frac{-b+\sqrt{b^{2}-4ac}}{2a}\right]}^{+},\rho_{c}^{l}\right\},\;\mathrm{if}\;g_{te}\geq g_{tr}\\ \min\left\{{\left[\frac{-b+\sqrt{b^{2}-4ac}}{2a}\right]}^{+},\rho_{T}\right\},\;\mathrm{if}\;g_{te}<g_{tr} \end{array}\;,\right.\mathrm{and}\;\rho_{3}=\left\{\begin{array}{c} \min\left\{{\left[\frac{-b-\sqrt{b^{2}-4ac}}{2a}\right]}^{+},\rho_{c}^{l}\right\},\;\mathrm{if}\;g_{te}\geq g_{tr}\\ \min\left\{{\left[\frac{-b-\sqrt{b^{2}-4ac}}{2a}\right]}^{+},\rho_{T}\right\},\;\mathrm{if}\;g_{te}<g_{tr} \end{array}\right. \end{array}$$

*in which a=1, $b=-\frac{\left[3\rho_{T}g_{te}+\left(2-\alpha \right)\rho_{J}g_{je}+3\right]g_{tr}-g_{te}}{2g_{tr}g_{te}}$, and $c=\frac{\left(\rho_{T}g_{tr}g_{te}+g_{tr}-g_{te}+\alpha g_{te}\right)\left(\rho_{T}g_{te}+\rho_{J}g_{je}+1\right)+\alpha g_{te}\left(\rho_{T}g_{te}+1\right)}{2g_{tr}g_{te}^{2}}$. It is true that $\sqrt{b^{2}-4ac}\geq 0$, and the proof is given as:*

**Proof.**  Define $f\left(\alpha \right)=b^{2}-4ac$. With some mathematic manipulations, we can obtain:

(5) $$\begin{array}{c} f\left(\alpha \right)=\frac{{\left(2-\alpha \right)}^{2}\rho_{J}^{2}g_{tr}^{2}g_{je}^{2}+\left(4-6\alpha \right)\rho_{J}g_{tr}g_{je}\left(\rho_{T}g_{tr}g_{te}+g_{tr}+g_{te}\right)+{\left(\rho_{T}g_{tr}g_{te}+g_{tr}+g_{te}\right)}^{2}}{4g_{tr}^{2}g_{te}^{2}}. \end{array}$$

Based on the property of the derivative, it is straightforward to know that f(α) is a decreasing function w.r.t. α for α∈[0,1], and $\min_{\begin{array}{c} \alpha \end{array}}f\left(\alpha \right)=f{\left(\alpha \right)}_{\alpha =1}\geq 0$. □

**Proof.**  The proof of Theorem 1 is presented in Appendix A. □

Notice that the one yielding the larger expected secrecy rate is regarded as $\rho_{c}^{⋆}$ between $\rho_{2}$ and $\rho_{3}$; meanwhile, the optimal transmit SNR of artificial interference is given by $\rho_{i}^{⋆}=\rho_{T}-\rho_{c}^{⋆}$. To get a better insight into the relationship between the optimal transmit SNR of the confidential message, $\rho_{c}^{⋆}$, and trust degree α, we have the following corollary.

**Corollary** **1.** *When the transmit SNR of the jamming signal goes to infinity, i.e., $\rho_{J}\to +\infty$, the optimal transmit SNR of the confidential message, $\rho_{c}^{⋆}$, can be obtained as:*

(6) $$\begin{array}{c} \rho_{c}^{⋆}=\left\{\begin{array}{cc} 0, & \mathrm{if}\;g_{te}\geq g_{tr},\;0\leq \alpha <\alpha_{1}\\ \rho_{\alpha }, & \mathrm{if}\;\left\{\begin{array}{c} g_{te}\geq g_{tr},\;\alpha_{1}\leq \alpha <\alpha_{2}\\ g_{te}<g_{tr},\;0\leq \alpha <\alpha_{3} \end{array}\right.\\ \rho_{c}^{l}, & \mathrm{if}\;\;\;g_{te}\geq g_{tr},\;\alpha_{2}\leq \alpha <\alpha_{3}\\ \rho_{T}, & \mathrm{if}\;\left\{\begin{array}{c} g_{te}\geq g_{tr},\\ g_{te}<g_{tr}, \end{array}\right.\alpha_{3}\leq \alpha \leq 1 \end{array}\right., \end{array}$$

*where $\rho_{\alpha }=\frac{\rho_{T}g_{tr}g_{te}+g_{tr}-g_{te}+\alpha g_{te}}{\left(2-\alpha \right)g_{tr}g_{te}}$, $\alpha_{1}={\left[\frac{g_{te}-g_{tr}-\rho_{T}g_{tr}g_{te}}{g_{te}}\right]}^{+}$, $\alpha_{2}={\left[\frac{\rho_{T}g_{tr}g_{te}+g_{tr}-g_{te}}{\rho_{T}g_{tr}g_{te}+g_{tr}}\right]}^{+}$, and $\alpha_{3}={\left[\frac{\rho_{T}g_{tr}g_{te}+g_{te}-g_{tr}}{\rho_{T}g_{tr}g_{te}+g_{te}}\right]}^{+}$.*

**Proof.**  The proof is given in Appendix C. □

**Remark** **1.** *From Corollary 1, we observe that the optimal transmit SNR of the confidential message, $\rho_{c}^{⋆}$, is mainly determined by the relationship among the trust degree, channel qualities, and the transmit SNR at T. If the eavesdropping channel quality is better than that of the legitimate channel such that $g_{te}\geq g_{tr}$, it is not secure to transmit any confidential message when α is small. With growing α within a moderate range, the transmit SNR of the confidential message basically depends on α. More specifically, T will reduce the power for artificial interference and allocate more power for the confidential message to exploit the cooperation of J fully. Furthermore, when J helps to jam with high probability, equivalently α is high, T relies on J to guarantee secure transmission and assigns all the power for confidential message. Notice that when the legitimate channel quality is good such that $g_{te}<g_{tr}$, it is still secure to send the confidential message with an appropriate transmit SNR even if α is small.*

## 4. Simulation Results

In this section, we evaluate the performance of the trust degree-based confidential cooperative communication and use the average gains of channel elements as $\left\{\sigma_{h_{tr}}^{2},\sigma_{h_{te}}^{2},\sigma_{h_{je}}^{2}\right\}=\left\{-20,-20,-20\right\}$ dB, and the expected secrecy rates are averaged over $10^{4}$ channel realizations.

**Figure 2:**
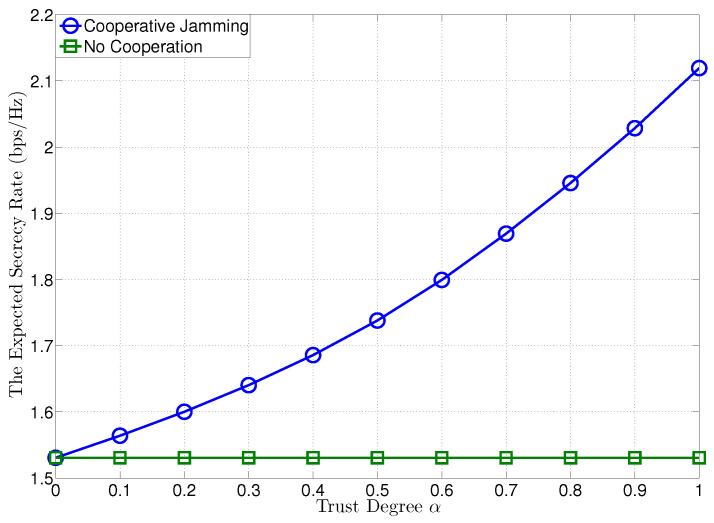
The expected secrecy rate versus trust degree α, where $\rho_{T}=\rho_{J}=30$ dB.

In Figure 2, we plot the expected secrecy rates at R versus the trust degree of J, when the transmit SNRs at T and J are given by $\rho_{T}=\rho_{J}=30$ dB. To compare with the proposed confidential cooperation scheme, we also plot the expected secrecy rate of the *no cooperation case* (NCC) (α=0). For the proposed confidential cooperation, the optimal transmit SNR of the confidential message at T for cooperation is obtained as $\rho_{c}^{⋆}$ in Theorem 1. In Figure 2, it is certified that the expected secrecy rate can be significantly improved by the jammer’s cooperation and increased with growing the trust degree of J, yielding a higher expected secrecy rate than NCC.

**Figure 3:**
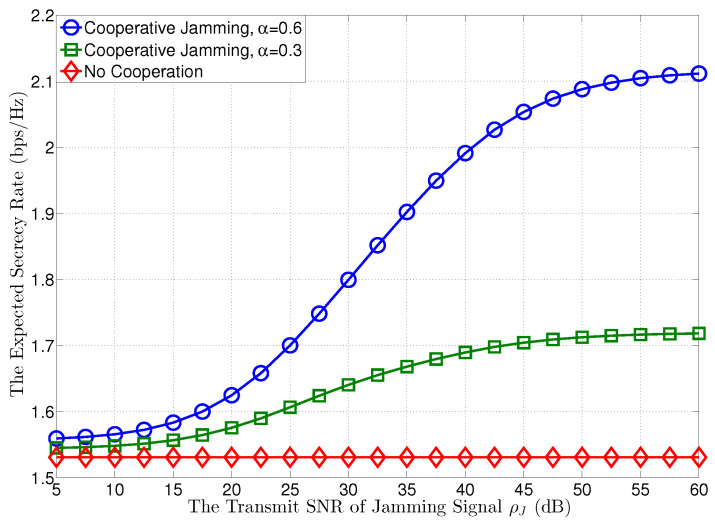
The expected secrecy rate versus the transmit SNR of jamming signal $\rho_{J}$, where $\rho_{T}=30$ dB.

In Figure 3, we plot the expected secrecy rates at R versus the transmit SNR of the jamming signal at J, when the transmit SNR at T is given by $\rho_{T}=30$ dB. In this figure, we first observe that when the transmit SNR of the jamming signal increases from 5 dB to 60 dB, i.e., $\rho_{J}$ goes to infinity, as shown in Corollary 1, the expected secrecy rate at R can be increased through the cooperation of J, and T will assign more power for the confidential message with growing α to achieve a higher expected secrecy rate. However, the expected secrecy rate does not increase all the time and will become saturated with the growth of $\rho_{J}$, as shown in (A5), which has no relationship with $\rho_{J}$ when $\rho_{J}$ goes to infinity.

## 5. Conclusions

In this paper, we investigated the confidential cooperative communication according to the jammer’s trust degree and exploited its characteristics to design efficient cooperative strategies. To maximize the expected secrecy rate, we derived the closed-form optimal transmit SNR of the confidential message based on the trust degree and observed that the expected secrecy rate will become saturated along with the increasing $\rho_{J}$. Simulation results showed that T will achieve a better performance with more power assignment for the confidential message instead of artificial interference with the growing trust degree of J, which has a great influence on the performance of confidential cooperation in terms of the expected secrecy rate.

## Appendix A. Proof of Theorem 1

If $g_{te}\geq g_{tr}$, from (3), we first deal with the operation of ${\left[x\right]}^{+}$ in (3) and consider two conditions (i) $\rho_{c}\leq \rho_{c}^{l}≜\frac{\rho_{T}g_{tr}g_{te}+g_{tr}-g_{te}}{g_{tr}g_{te}}$ and (ii) $\rho_{c}\leq \rho_{c}^{u}≜\frac{\rho_{T}g_{tr}g_{te}+\rho_{J}g_{tr}g_{je}+g_{tr}-g_{te}}{g_{tr}g_{te}}$ to make the first and the second term in (3) non-negative. Notice that $\rho_{c}\geq 0$ and $\rho_{J}\geq 0$. According to (i) and (ii), we investigate (3) and solve P as follows:

(1) If $\rho_{c}^{u}\leq \rho_{c}\leq \rho_{T}$, we obtain $\rho_{J}\leq \frac{g_{te}-g_{tr}}{g_{tr}g_{je}}$, and ${\bar{R}}_{se}\left(\rho_{c}\right)=0$.

(2) If $\rho_{c}^{l}\leq \rho_{c}<\rho_{c}^{u}\leq \rho_{T}$,

(A1) R¯se(ρc)=αlog21+ρcgte(ρT−ρc)gte+ρJgje+1log2(1+ρcgtr)−log21+ρcgte(ρT−ρc)gte+ρJgje+1,

which is a concave function w.r.t. $\rho_{c}$. Resorting to $\frac{d{\bar{R}}_{se}\left(\rho_{c}\right)}{d\rho_{c}}=0$, we can get $\rho_{c}^{o}=\frac{\rho_{T}g_{tr}g_{te}+\rho_{J}g_{tr}g_{je}+g_{tr}-g_{te}}{2g_{tr}g_{te}}$. Based on whether $\rho_{c}^{o}$ lies in $\left[\rho_{c}^{l},\;\rho_{c}^{u}\right)$, different situations are given as follows:

- If ${\left[\frac{\rho_{T}g_{tr}g_{te}+g_{tr}-g_{te}}{g_{tr}g_{je}}\right]}^{+}\leq \rho_{J}\leq \frac{g_{te}-g_{tr}}{g_{tr}g_{je}}$, $\rho_{c}^{o}\in \left[\rho_{c}^{l},\;\rho_{c}^{u}\right)$, the optimal transmit SNR of the confidential message that maximizes (A1) is obtained by $\rho_{c}^{⋆}=\frac{\rho_{T}g_{tr}g_{te}+\rho_{J}g_{tr}g_{je}+g_{tr}-g_{te}}{2g_{tr}g_{te}}$;
- If $\rho_{J}\leq \min\left\{{\left[\frac{\rho_{T}g_{tr}g_{te}+g_{tr}-g_{te}}{g_{tr}g_{je}}\right]}^{+},\frac{g_{te}-g_{tr}}{g_{tr}g_{je}}\right\}$, $\rho_{c}^{o}\in \left[0,\;\rho_{c}^{l}\right]$, the optimal transmit SNR of the confidential message is obtained by $\rho_{c}^{⋆}=\rho_{c}^{l}$ due to the concavity of (A1).

(3) If $0\leq \rho_{c}\leq \rho_{c}^{l}\leq \rho_{c}^{u}\leq \rho_{T}$ or $0\leq \rho_{c}\leq \rho_{c}^{l}\leq \rho_{T}\leq \rho_{c}^{u}$, where the boundary point $\rho_{c}^{l}$ can be included in this interval without affecting the optimal transmit SNR of confidential message $\rho_{c}^{⋆}$, the formulation of ${\bar{R}}_{se}\left(\rho_{c}\right)$ is the same, given by:

(A2) $${\bar{R}}_{se}\left(\rho_{c}\right)=\log_{2}\left(1+\rho_{c}g_{tr}\right)-\bar{\alpha }\log_{2}\left(1+\frac{\rho_{c}g_{te}}{\left(\rho_{T}-\rho_{c}\right)g_{te}+1}\right)-\alpha \log_{2}\left(1+\frac{\rho_{c}g_{te}}{\left(\rho_{T}-\rho_{c}\right)g_{te}+\rho_{J}g_{je}+1}\right),$$

which is concave w.r.t. $\rho_{c}$, and the proof is given in Appendix B. With the help of factorization, the optimal transmit SNR of the confidential message that maximizes (A2) is obtained by using $\frac{d{\bar{R}}_{se}\left(\rho_{c}\right)}{d\rho_{c}}=0$ to get $\rho_{c}^{o}$, which is part of $\rho_{2}$ and $\rho_{3}$ in Theorem 1. Notice that it is necessary to check the feasibility of $\rho_{c}^{o}$ whether it lies in $\left[0,\;\rho_{c}^{l}\right]$. If not, the boundary point is chosen as $\rho_{c}^{⋆}$ due to the concavity of (A2), and thus, the result in the second part of (2) in this Appendix can be classified in this part.

(4) If $\rho_{c}^{l}\leq \rho_{c}\leq \rho_{T}<\rho_{c}^{u}$, we obtain $\rho_{J}>\frac{g_{te}-g_{tr}}{g_{tr}g_{je}}$, and the formulation of ${\bar{R}}_{se}\left(\rho_{c}\right)$ is given by (A1). Resorting to $\frac{d{\bar{R}}_{se}\left(\rho_{c}\right)}{d\rho_{c}}=0$, we can get $\rho_{c}^{o}=\frac{\rho_{T}g_{tr}g_{te}+\rho_{J}g_{tr}g_{je}+g_{tr}-g_{te}}{2g_{tr}g_{te}}$. Based on the feasibility check, we can get the following results:

- If $\frac{\rho_{T}g_{tr}g_{te}+g_{te}-g_{tr}}{g_{tr}g_{je}}<\rho_{J}$, $\rho_{c}^{o}>\rho_{T}$, the optimal transmit SNR of the confidential message is obtained by $\rho_{c}^{⋆}=\rho_{T}$ due to the concavity of (A1);
- If $\max\left\{\frac{g_{te}-g_{tr}}{g_{tr}g_{je}},\frac{\rho_{T}g_{tr}g_{te}+g_{tr}-g_{te}}{g_{tr}g_{je}}\right\}<\rho_{J}\leq \frac{\rho_{T}g_{tr}g_{te}+g_{te}-g_{tr}}{g_{tr}g_{je}}$, $\rho_{c}^{o}\in \left(\rho_{c}^{l},\;\rho_{T}\right]$, the optimal transmit SNR of the confidential message is obtained by $\rho_{c}^{⋆}=\frac{\rho_{T}g_{tr}g_{te}+\rho_{J}g_{tr}g_{je}+g_{tr}-g_{te}}{2g_{tr}g_{te}}$;
- If $\frac{g_{te}-g_{tr}}{g_{tr}g_{je}}<\rho_{J}\leq \frac{\rho_{T}g_{tr}g_{te}+g_{tr}-g_{te}}{g_{tr}g_{je}}$, $\rho_{c}^{o}\in \left[0,\;\rho_{c}^{l}\right]$, the optimal transmit SNR of the confidential message is $\rho_{c}^{⋆}=\rho_{c}^{l}$, and this result can be categorized into (3) in this Appendix.

If $g_{te}<g_{tr}$, the formulation of ${\bar{R}}_{se}\left(\rho_{c}\right)$ is given in (A2) with $0\leq \rho_{c}\leq \rho_{T}<\rho_{c}^{l}$, and the optimal transmit SNR of the confidential message is similar to that in (3).

## Appendix B. Proof of the Concavity of (A2)

The following proof is to show the concavity of (A2). We first obtain the derivative of (A2) w.r.t. $\rho_{c}$ as:

(A3) $$\frac{d{\bar{R}}_{se}\left(\rho_{c}\right)}{d\rho_{c}}=\frac{1}{\ln2}\left(\frac{g_{tr}}{1+\rho_{c}g_{tr}}-\frac{\bar{\alpha }g_{te}}{\rho_{T}g_{te}-\rho_{c}g_{te}+1}-\frac{\alpha g_{te}}{\rho_{T}g_{te}-\rho_{c}g_{te}+\rho_{J}g_{je}+1}\right).$$

To know whether (A2) is concave or not w.r.t $\rho_{c}$, we further obtain its second derivative as:

(A4) $$\frac{d^{2}{\bar{R}}_{se}\left(\rho_{c}\right)}{d\rho_{c}^{2}}=\frac{-1}{\ln2}\left[\frac{g_{tr}^{2}}{{\left(1+\rho_{c}g_{tr}\right)}^{2}}+\frac{\bar{\alpha }g_{te}^{2}}{{\left(\rho_{T}g_{te}-\rho_{c}g_{te}+1\right)}^{2}}+\frac{\alpha g_{te}^{2}}{{\left(\rho_{T}g_{te}-\rho_{c}g_{te}+\rho_{J}g_{je}+1\right)}^{2}}\right].$$

Based on the expression of $\frac{d^{2}{\bar{R}}_{se}\left(\rho_{c}\right)}{d\rho_{c}^{2}}$ above, the second derivative is negative, and thus, (A2) is concave w.r.t. $\rho_{c}$ [25].

## Appendix C. Proof of Corollary 1

When $\rho_{J}\to +\infty$, we only need to consider Case (3), Case (4), and the case when $g_{te}<g_{tr}$ in Appendix A. If $g_{te}\geq g_{tr}$, (3) is recast as follows:

(1) If $0\leq \rho_{c}\leq \rho_{c}^{l}\leq \rho_{T}\ll \rho_{c}^{u}$,

(A5) $${\bar{R}}_{se}\left(\rho_{c}\right)=\log_{2}\left(1+\rho_{c}g_{tr}\right)-\bar{\alpha }\log_{2}\left(1+\frac{\rho_{c}g_{te}}{\left(\rho_{T}-\rho_{c}\right)g_{te}+1}\right),$$

which is concave w.r.t. $\rho_{c}$. Resorting to $\frac{d{\bar{R}}_{se}\left(\rho_{c}\right)}{d\rho_{c}}=0$, we have $\rho_{c}^{o}=\frac{\rho_{T}g_{tr}g_{te}+g_{tr}-g_{te}+\alpha g_{te}}{\left(2-\alpha \right)g_{tr}g_{te}}$. Based on whether $\rho_{c}^{o}$ lies in $\left[0,\;\rho_{c}^{l}\right]$, different situations are given as:

- If $0\leq \alpha <{\left[\frac{g_{te}-g_{tr}-\rho_{T}g_{tr}g_{te}}{g_{te}}\right]}^{+}$, $\rho_{c}^{o}<0$, the optimal transmit SNR of the confidential message is $\rho_{c}^{⋆}=0$ due to the concavity of (A5);
- If ${\left[\frac{g_{te}-g_{tr}-\rho_{T}g_{tr}g_{te}}{g_{te}}\right]}^{+}\leq \alpha <{\left[\frac{\rho_{T}g_{tr}g_{te}+g_{tr}-g_{te}}{\rho_{T}g_{tr}g_{te}+g_{tr}}\right]}^{+}$, $\rho_{c}^{o}\in \left[0,\;\rho_{c}^{l}\right)$, the optimal transmit SNR of the confidential message that maximizes (A5) is $\rho_{c}^{⋆}=\frac{\rho_{T}g_{tr}g_{te}+g_{tr}-g_{te}+\alpha g_{te}}{\left(2-\alpha \right)g_{tr}g_{te}}$;
- If ${\left[\frac{\rho_{T}g_{tr}g_{te}+g_{tr}-g_{te}}{\rho_{T}g_{tr}g_{te}+g_{tr}}\right]}^{+}\leq \alpha <{\left[\frac{\rho_{T}g_{tr}g_{te}+g_{te}-g_{tr}}{\rho_{T}g_{tr}g_{te}+g_{te}}\right]}^{+}$, $\rho_{c}^{o}\in \left[\rho_{c}^{l},\;\rho_{T}\right]$, the optimal transmit SNR of the confidential message is $\rho_{c}^{⋆}=\rho_{c}^{l}$ due to the concavity of (A5).

(2) If $\rho_{c}^{l}\leq \rho_{c}\leq \rho_{T}\ll \rho_{c}^{u}$, where the boundary point $\rho_{c}^{l}$ can be included in this interval without affecting the optimal transmit SNR of confidential message $\rho_{c}^{⋆}$, ${\bar{R}}_{se}\left(\rho_{c}\right)=\alpha \log_{2}\left(1+\rho_{c}g_{tr}\right)$, and the optimal transmit SNR of the confidential message is $\rho_{c}^{⋆}=\rho_{T}$.

If $g_{te}<g_{tr}$, the formulation of ${\bar{R}}_{se}\left(\rho_{c}\right)$ is the same as (A5) with $0\leq \rho_{c}\leq \rho_{T}$. Due to its concavity w.r.t. $\rho_{c}$, we can get $\rho_{c}^{o}=\frac{\rho_{T}g_{tr}g_{te}+g_{tr}-g_{te}+\alpha g_{te}}{\left(2-\alpha \right)g_{tr}g_{te}}$ resorting to $\frac{d{\bar{R}}_{se}\left(\rho_{c}\right)}{d\rho_{c}}=0$. Based on whether $\rho_{c}^{o}$ lies in $\left[0,\;\rho_{T}\right]$, the following different situations are given as:

- If $0\leq \alpha <{\left[\frac{\rho_{T}g_{tr}g_{te}+g_{te}-g_{tr}}{\rho_{T}g_{tr}g_{te}+g_{te}}\right]}^{+}$, $\rho_{c}^{o}\in \left[0,\;\rho_{T}\right]$, the optimal transmit SNR of the confidential message is $\rho_{c}^{⋆}=\frac{\rho_{T}g_{tr}g_{te}+g_{tr}-g_{te}+\alpha g_{te}}{\left(2-\alpha \right)g_{tr}g_{te}}$;
- If ${\left[\frac{\rho_{T}g_{tr}g_{te}+g_{te}-g_{tr}}{\rho_{T}g_{tr}g_{te}+g_{te}}\right]}^{+}\leq \alpha \leq 1$, $\rho_{c}^{o}>\rho_{T}$, the optimal transmit SNR of the confidential message is $\rho_{c}^{⋆}=\rho_{T}$ due to the concavity of (A5).
